# Tricuspid Structural Valve Deterioration Treated with a Transcatheter Valve-in-Valve Implantation: A Single-Center Prospective Registry

**DOI:** 10.3390/jcm11092667

**Published:** 2022-05-09

**Authors:** Nili Schamroth Pravda, Hana Vaknin Assa, Amos Levi, Guy Witberg, Yaron Shapira, Mordechai Vaturi, Katia Orvin, Yeela Talmor Barkan, Ashraf Hamdan, Raffael Mishaev, Ram Sharoni, Leor Perl, Alexander Sagie, Ran Kornowski, Pablo Codner

**Affiliations:** 1 Department of Cardiology, Rabin Medical Center, Petach Tikva 4941492, Israel; hana100niki@gmail.com (H.V.A.); amos.levi@gmail.com (A.L.); vitberguy@gmail.com (G.W.); yarons2@clalit.org.il (Y.S.); vaturim@gmail.com (M.V.); katiaorvin@gmail.com (K.O.); talmor.yeela@gmail.com (Y.T.B.); hamdashraf@gmail.com (A.H.); leorperl@gmail.com (L.P.); alik.sagie2@gmail.com (A.S.); ran.kornowski@gmail.com (R.K.); drpablocodner@gmail.com (P.C.); 2 Faculty of Medicine, Tel Aviv University, Tel Aviv 6423906, Israel; raffmishaev@gmail.com (R.M.); ramsh@clalit.org.il (R.S.); 3 Cardio-Thoracic Surgery Department, Rabin Medical Center, Petach Tikva 4941492, Israel

**Keywords:** tricuspid valve, structural valve deterioration, valve-in-valve, transcatheter, outcomes

## Abstract

The valve-in-valve (ViV) technique is an emerging alternative for the treatment of bioprosthetic structural valve deterioration (SVD) in the tricuspid position. We report on the outcomes of patients treated by a transcatheter tricuspid valve-in-valve (TT-ViV) implantation for symptomatic SVD in the tricuspid position during the years 2010–2019 at our center. Three main outcomes were examined during the follow-up period: TT-ViV hemodynamic data per echocardiography, mortality and NYHA functional class. Our cohort consisted of 12 patients with a mean age 65.4 ± 11.9 years, 83.3% male. The mean time from initial valve intervention to TT-ViV was 17.4 ± 8.7 years. The indications for TT-ViV were varied (41.7% for predominant regurgitation, 33.3% for predominant stenosis and 25.0% with a mixed pathology). All patients were treated with a balloon-expandable device. The mean follow-up was 3.4 ± 1.3 years. Tricuspid regurgitation was ≥ moderate in 57.2% of patients prior to the procedure and this decreased to 0% following the procedure. The mean transtricuspid valve gradients mildly decreased from the mean pre-procedural values of 9.0 mmHg to 7.0 mmHg at one month following the procedure (*p* = 0.36). Mortality at one year was 8.0% (95% CI 0–23). At the baseline, 4 patients (33.3%) were in NYHA functional class III/IV; this was reduced to 2 patients (18.2%) at the one year follow-up and both were in NYHA III. The TT-ViV procedure offered a safe, feasible and less invasive treatment option for patients with SVD in our detailed cohort.

## 1. Introduction

There is an increasing number of patients presenting with structural valve deterioration (SVD) following the increasing use of bioprosthetic surgical valve replacements for the treatment of native valve disease. Transcatheter valve interventions are becoming an increasingly validated treatment option in these patients [1]. There are limited data on the clinical outcomes of patients undergoing valve-in-valve implantations in the tricuspid position [2,3]. We report on our clinical experience of treating patients using the valve-in-valve (ViV) technique in the tricuspid position at our institution over the intermediate-term.

## 2. Materials and Methods

The characteristics and outcomes of patients with bioprosthetic SVD treated by the implantation of a transcatheter tricuspid valve-in-valve (TT-ViV) device within a failed surgical valve are described in the present report. The cohort included patients undergoing TT-ViV procedures performed from February 2014 to June 2018. The patient data follow-up was completed in November 2021. The operative risk was determined by the logistic European System for Cardiac Operative Risk Evaluation score (log EUROSCORE) and the score of the Society of Thoracic Surgeons (STS). All patients underwent transthoracic and transesophageal (TEE) echocardiograms as part of the initial workup. The transcatheter heart valve sizes were assessed based on these measurements. Gated cardiac computer tomography was performed as an additive imaging tool on an individual basis. During the procedure, different stiff guidewires were deployed at the right ventricle or main pulmonary artery to provide support for the transcatheter heart valves. This was dependent on individual patient characteristics. The transcatheter heart valves were positioned within the prior bioprosthesis valve with TEE and fluoroscopic guidance.

The baseline, procedural and peri-procedural findings were described. The prospective data collection was approved by the institutional review board. Three endpoints were examined: NYHA (New York Heart Association) functional status at one year; valve hemodynamics of the implanted valves as per the echocardiography performed at one month after the procedure and yearly thereafter; and the rates of survival during the follow-up period.

The data on mortality were based on mortality files derived from the notification of death form legally required by the Ministry of the Interior. Follow-up data were available for eleven patients at the one year follow-up (one died in this period). The baseline characteristics of the patients were presented as a mean and standard deviation (SD) for the continuous variables and the count (%) for the categorical variables. The continuous variables were compared using the Student’s *t*-test/Mann–Whitney U test and the categorical variables were compared using the chi-squared/Fisher’s exact test, as appropriate. All tests were two-tailed and a *p*-value < 0.05 was considered to be significant. All-cause mortality was graphically plotted using Kaplan–Meier curves and compared between the groups using the log rank test (unadjusted analysis). All TT-ViV-related data were registered in an electronic file and analyzed using R (R-studio, V.4.0.0, Vienna, Austria).

## 3. Results

The baseline clinical and echocardiographic characteristics of the cohort are shown in Table 1. Our cohort consisted of 12 patients with a mean age 65.4 ± 11.9 years, 83.3% male. The indications for TT-ViV were varied (41.7% for predominant regurgitation, 33.3% for predominant stenosis and 25% with a mixed pathology). The mean time from the initial valve intervention to TT-ViV was 17.4 ± 8.7 years. Three patients had more than one prior tricuspid valve surgical intervention (two had re-do surgical tricuspid valve replacements (TVR) and one had undergone a tricuspid commissurotomy and a subsequent tricuspid valve replacement). Of the 12 patients, 10 had other concomitant valvular diseases (7 had a mechanical mitral valve and 3 had aortic valve disease), 1 patient had an Ebstein anomaly of the tricuspid valve and 1 patient had a tricuspid valve pathology secondary to infective endocarditis. Two patients were adults with congenital heart disease; one had an Ebstein anomaly (as previously mentioned) and one had undergone previous surgery for a discrete subaortic membrane and Ross–Kono valve surgery.

All patients were treated with balloon-expandable, transcatheter heart valves: Sapien 3 (*n* = 11) and Sapien XT™ (*n* = 1) (Edwards Lifesciences, Irvine, CA, USA). The procedural characteristics are shown in Table 2. The list of the type and size of the bioprosthetic valves and the corresponding transcatheter valve devices is shown in Table 3. The mean follow-up was 3.4 ± 1.3 years. The average hospital stay was 4.1 ± 2.8 days.

### 3.1. Hemodynamic Parameters

**Figure 1:**
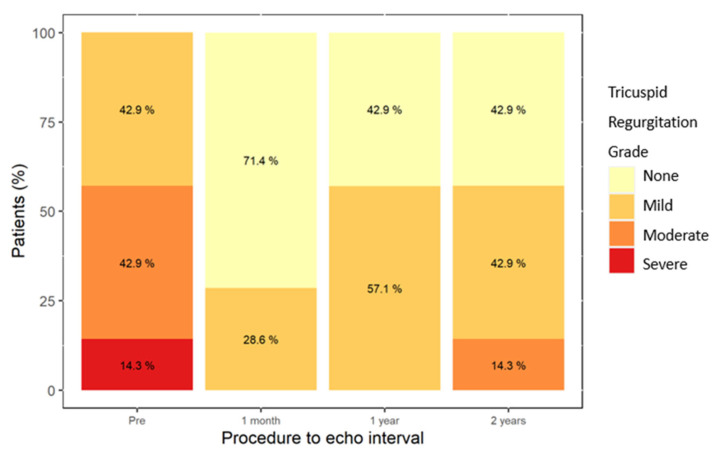
Severity of tricuspid regurgitation during follow-up.

The temporal changes in the hemodynamic indexes were assessed by echocardiography. Tricuspid regurgitation was ≥moderate in 57.2% of patients prior to the procedure and this decreased to 0% following the procedure (Figure 1).

**Figure 2:**
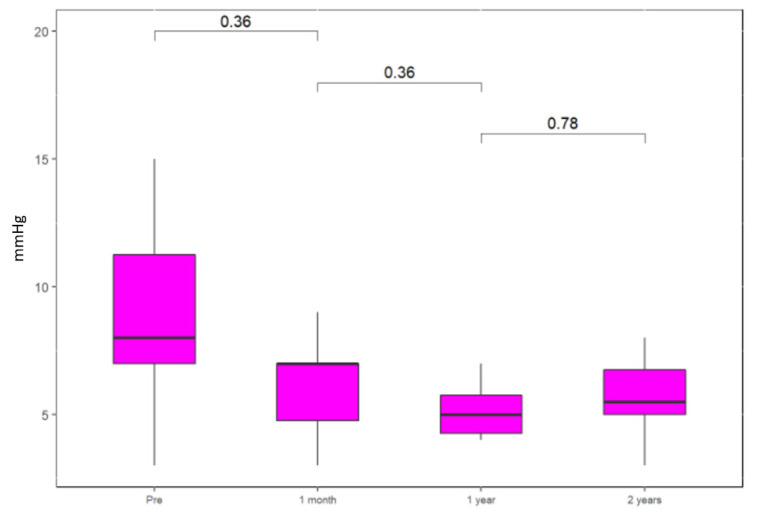
Mean gradients across tricuspid valve.

The mean tricuspid valve gradients decreased slightly from the pre-procedural values of 9.0 mmHg (95% CI 7.0; 12.0) to 7.0 (95% CI 5.0; 7.5) at one month following the procedure (*p* = 0.36) (Figure 2). The gradients remained steady during the follow-up. There were mild or no right ventricular dysfunction measures in all patients in our cohort and this did not change over the follow-up period. This is shown in Supplementary Figure S1.

**Figure 3:**
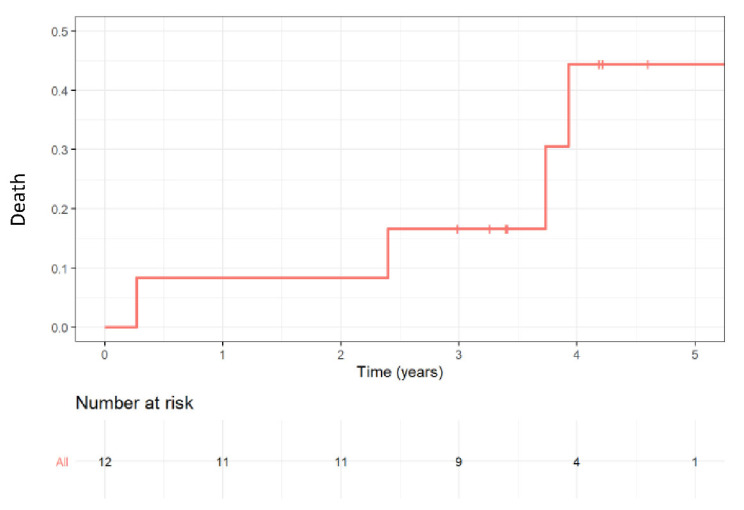
Kaplan–Meier mortality curve during follow-up.

Mortality rates at the one year follow-up were 8.0%, as shown in Figure 3. Two patients died due to infectious causes; one patient from malignancy and the cause of death was unknown in one patient. One patient had a valve thrombosis 30 days following the procedure and was medically managed using anticoagulation. There were two patients with minor bleeding following the procedure and one patient who developed a stage 1 acute kidney injury.

### 3.2. Functional Status

**Figure 4:**
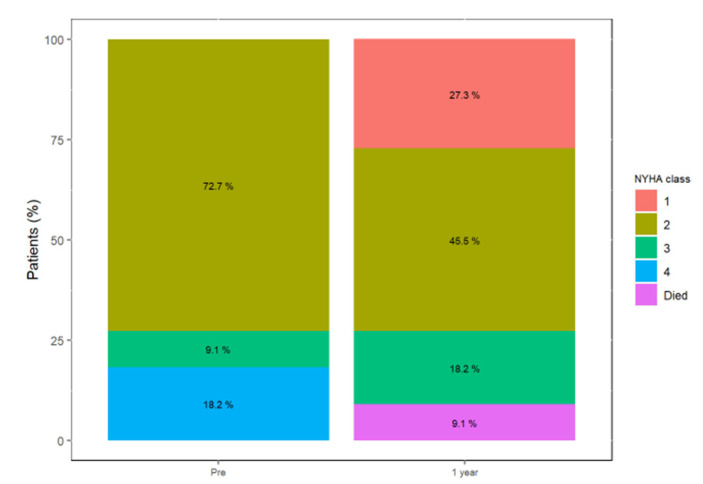
NYHA functional status at baseline and one year follow-up.

At the baseline, four patients (33.3%) were in NYHA functional class III/IV; this was reduced to two patients (18.2%) at the one year follow-up and both patients had an NYHA III status. This is shown in Figure 4.

## 4. Discussion

The main objective of our study was to report on the clinical outcomes of patients with SVD treated with TT-ViV at our center. Our findings from this retrospective study demonstrated the following: the functional class of the patients improved following TT-ViV; the hemodynamic response to the procedure was favorable; and mortality was low and largely secondary to non-cardiac causes. Isolated tricuspid valve surgery carries a high morbidity and the highest mortality of valve surgeries and, as such, is a high-risk subgroup and is rarely performed [4,5,6]. Re-do tricuspid surgery carries an even higher risk, mostly due to the complex clinical milieu of the disease. Transcatheter valve repairs and replacements are evolving as a less invasive alternative to surgery and are an appealing option to those needing a repeat intervention [7]. The tricuspid valve is saddle-shaped and this unique anatomy can be challenging in transcatheter valve procedures. There is increasing evidence supporting transcatheter tricuspid valve replacements as a treatment option in those with a severe tricuspid regurgitation and a high surgical risk. Lu et al. recently published promising early results showing a significant reduction in tricuspid regurgitation and low complication rates [8].

Our report adds to an increasing body of evidence supporting TT-ViV as a treatment option in patients with SVD [9]. Our results are similar to those reported in the international VIVID registry (Valve-in-Valve International Database Registry) [3,10]. This registry compiled data on 306 patients from 80 centers that underwent TT-ViV. The mid-term outcomes at a median follow-up of 15.9 months showed that TT-ViV offered a clinical and hemodynamic improvement. During the follow-up period, 36 patients (11.7% of the cohort) died. This finding was similar to our cohort with a low one-year mortality. It is important to note that although the cohort was relatively young, with a median age of 40 years, the majority of patients were NYHA III/IV at the time of the procedure and NYHA IV was significantly associated with an increased hazard ratio of death (HR 2.9; 95% CI 1.2–7.3; *p* = 0.021). This was unlike our cohort, who—although older—were mostly NYHA I/II at the baseline. This reflects the importance of performing an early intervention in the SVD process at our institution. Another important hemodynamic factor in our cohort was that of a right ventricular dysfunction. All our patients had mild or no right ventricular (RV) dysfunctions and this did not change over the follow-up period. The early timing of the intervention is central to prevent irreversible RV damage; this is pivotal not only to the TT-ViV function, but also to the clinical outcomes [6]. The hemodynamics of TT-ViV during the follow-up were favorable and no patient developed a significant residual or recurrent TT-ViV dysfunction during the follow-up, defined by the VIVID authors as a tricuspid mean gradient ≥ 10 mmHg [10]. The study limitations were that this was a single-center retrospective analysis and a small cohort. There was an inherent selection bias of our cohort because all patients underwent a thorough assessment process as candidates for this procedure. This was evident as the average STS score was intermediate (and not very high risk) These factors may partially explain our encouraging outcomes. Our center has a dedicated structural percutaneous interventional unit. Our cohort was small, but this study is novel in that it is one of the largest single-center reports on TT-ViV. The authors of the VIVID registry, the largest registry of its kind, remarked that most centers in the registry had experience with 1–3 procedures [10]. Another strength of our study was the quality of our data acquisition. We have a dedicated data collection team and a structured clinical and imaging follow-up program to ensure the careful acquisition and quality of our data. Although our results are encouraging, there is a need for further data in this field and the long-term function and durability of TT-ViV are still unknown.

## 5. Conclusions

TT-ViV is an emerging treatment option for SVD in the tricuspid position. In our single-center experience, TT-ViV for the treatment of SVD was safe and effective. These results are encouraging, but further data on long-term outcomes are needed.

## Figures and Tables

**Table 1:** Baseline characteristic of patients who underwent valve-in-valve implantation in the tricuspid position.

TT-ViV (*n* = 12)	Mean/Percentage
Age (years ± SD)	65.4 ± 11.9
Male (%)	10 (83.3)
BMI (units)	26.6 ± 7.1
STS	4.1 ± 3.1
Euroscore II	5.4 ± 3.4
Coronary artery disease (%)	1 (8.3)
Prior coronary artery bypass surgery (%)	0
Prior PCI (%)	1 (9.1)
Diabetes mellitus	6 (50)
Hypertension	7 (58.3)
Chronic dialysis	0 (0)
Chronic obstructive pulmonary disease	2 (16.7)
Atrial fibrillation/flutter	10 (83.3)
Permanent pacemaker/defibrillator	5 (41.7)
NYHA functional class III/IV	4 (33.3)
Hemoglobin (g/dL)	11.6 ± 2.3
GFR (MDRD)	62.6 ± 24.3
Albumin	4.2 ± 0.4
Systolic pulmonary artery pressure (mmHg)	24.5 ± 27.4
Size of valve treated (mm)	
27	2 (18.2)
29	2 (18.2)
31	2 (18.2)
33	5 (45.5)
LV systolic function	
Normal (>50%)	11 (91.7)
Mild (40–49%)	1 (8.3)
Moderate or more tricuspid regurgitation	4 (33.3)
Valve pathology	
Stenosis	4 (33.3)
Regurgitation	5 (41.7)
Combined	3 (25.0)

TT-ViV: transcatheter tricuspid valve-in-valve; BMI: body mass index; STS: Society of Thoracic Surgeons; PCI: percutaneous coronary intervention; CVA/TIA: cerebrovascular accident/transient ischemic attack; NYHA: New York Heart Association; GFR: glomerular filtration rate; MDRD: modification of diet in renal disease.

**Table 2:** Procedural characteristics: valve-in-valve tricuspid position.

TT-ViV (*n* = 12)	Number (%)
Urgent procedure, *n* (%)	1 (8.3)
Conscious sedation or local anesthesia only	7 (58.3)
General anesthesia	5 (41.5)
TEE guidance	7 (58.3)
Vascular access via femoral vein	12 (100)
Size of ViV used (mm)	
26	2 (16.7)
29	10 (83.3)
Balloon-expandable valve	12 (100)
Fluoroscopy time (min)	17.6 ± 14.8
Contrast volume (mL)	10.0 ± 14.1

**Table 3:** Bioprosthetic valve type and size and their corresponding transcatheter valve devices.

Tradename Surgical Valve	Surgical Valve Size	Number of Previous Tricuspid Interventions Prior to TT-ViV	TT-ViV Valve	TT-ViV Size
Xenograft	29	2	Edwards SAPIEN XT	29
Carpentier Edwards	33	1	Edwards SAPIEN 3	29
Hancock II	33	1	Edwards SAPIEN 3	29
Epic	33	1	Edwards SAPIEN 3	29
Hancock II	33	1	Edwards SAPIEN 3	29
Carpentier Edwards	27	1	Edwards SAPIEN 3	26
Hancock II 29	29	1	Edwards SAPIEN 3	29
Carpentier Edwards	31	1	Edwards SAPIEN 3	29
Carpentier Edwards	33	2	Edwards SAPIEN 3	29
Hancock II	27	1	Edwards SAPIEN 3	26
Carpentier Edwards	31	1	Edwards SAPIEN 3	29
Xenograft	Not known	2	Edwards SAPIEN 3	29

## Supplementary Materials

The following supporting information can be downloaded at: https://www.mdpi.com/article/10.3390/jcm11092667/s1, Figure S1: Right ventricular function during follow up.
