# Supplementary figures and images for: Tricuspid Structural Valve Deterioration Treated with a Transcatheter Valve-in-Valve Implantation: A Single-Center Prospective Registry
Source: J Clin Med. 2022 May 9;11(9):2667. doi: 10.3390/jcm11092667 (PMC9104146; doi:10.3390/jcm11092667)

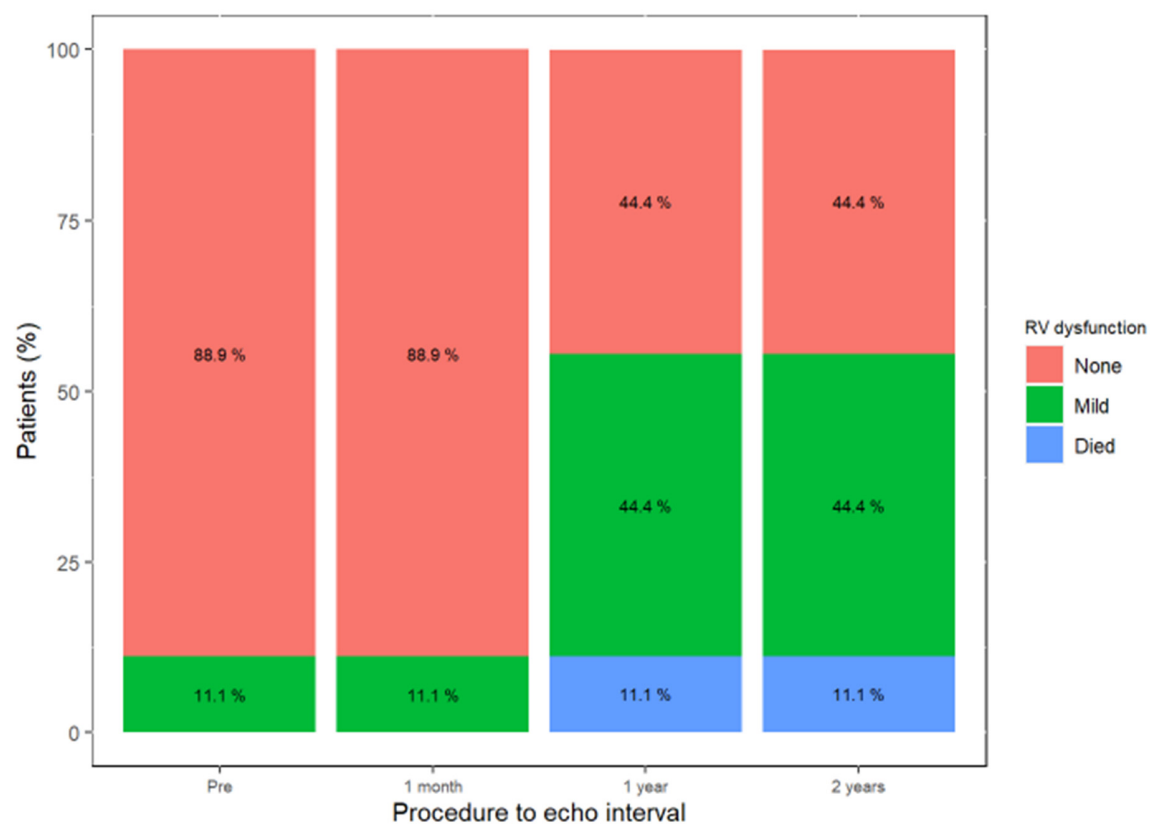

**Supplementary Figure S1.** Right ventricular function during follow up.

Supplement: Supplementary file 1 [file jcm-11-02667-s001.zip › jcm-1689458-supplementary.pdf]
